# Spindle Assembly Checkpoint of Oocytes Depends on a Kinetochore Structure Determined by Cohesin in Meiosis I

**DOI:** 10.1016/j.cub.2013.10.052

**Published:** 2013-12-16

**Authors:** Kikuë Tachibana-Konwalski, Jonathan Godwin, Máté Borsos, Ahmed Rattani, David J. Adams, Kim Nasmyth

**Affiliations:** 1 Institute of Molecular Biotechnology of the Austrian Academy of Sciences (IMBA), Dr. Bohr-Gasse 3, 1030 Vienna, Austria; 2 Department of Biochemistry, University of Oxford, South Parks Road, Oxford OX1 3QU, UK; 3 Wellcome Trust Sanger Institute, Hinxton, Cambridge CB10 1SA, UK

## Abstract

Since the dissolution of sister chromatid cohesion by separase and cyclin B destruction is irreversible, it is essential to delay both until all chromosomes have bioriented on the mitotic spindle. Kinetochores that are not correctly attached to the spindle generate the mitotic checkpoint complex (MCC), which inhibits the anaphase-promoting complex/cyclosome (APC/C) and blocks anaphase onset. This process is known as the spindle assembly checkpoint (SAC) [1]. The SAC is especially important in meiosis I, where bivalents consisting of homologous chromosomes held together by chiasmata biorient. Since the first meiotic division is unaffected by rare achiasmatic chromosomes or misaligned bivalents [2–7], it is thought that several tensionless kinetochores are required to produce sufficient MCC for APC/C inhibition. Consistent with this, univalents lacking chiasmata elicit a SAC-mediated arrest in *Mlh1*^−/−^ oocytes. In contrast, chromatids generated by TEV protease-induced cohesin cleavage in *Rec8*^TEV/TEV^ oocytes merely delay APC/C activation. Since the arrest of *Mlh1*^−/−^*Rec8*^TEV/TEV^ oocytes is alleviated by TEV protease, even when targeted to kinetochores, we conclude that their SAC depends on cohesin as well as dedicated kinetochore proteins. This has important implications for aging oocytes [8, 9], where cohesin deterioration will induce sister kinetochore biorientation and compromise MCC production, leading to chromosome missegregation and aneuploid fetuses.

## Highlights

• Cohesin is necessary for robust spindle assembly checkpoint activity in meiosis I
• Cohesin dependence of the spindle assembly checkpoint is meiosis specific
• Centromeric cohesion is necessary for sister kinetochore mono-orientation

## Results and Discussion

The spindle assembly checkpoint (SAC) is essential for delaying anaphase onset in meiosis I [10–16]. Many bivalents undergo one or more rounds of error correction before they achieve stable biorientation [17], as was initially observed in grasshopper spermatocytes [18]. It is presumed, but not known with certainty, that kinetochores of bivalents that have not yet bioriented fail to come under the tension required to turn off mitotic checkpoint complex (MCC) production. Consistent with this, oocytes from many but not all [19] strains of *Mlh1*^−/−^ mice, which cannot form chiasmata and accumulate up to 40 univalent chromosomes, rarely extrude polar bodies [20]. Because polar body extrusion (PBE) is unaffected by the presence of a single unpaired X chromosome ([2] and our unpublished data) or by rare achiasmatic chromosomes [3], it is thought that several “mono-oriented” kinetochores are required to produce sufficient MCC to inhibit the APC/C effectively. How MCC production is regulated by kinetochore structure is poorly understood.

### SAC Response to Kinetochores Associated with Chromatids in Meiosis I Oocytes

**Figure 1:**
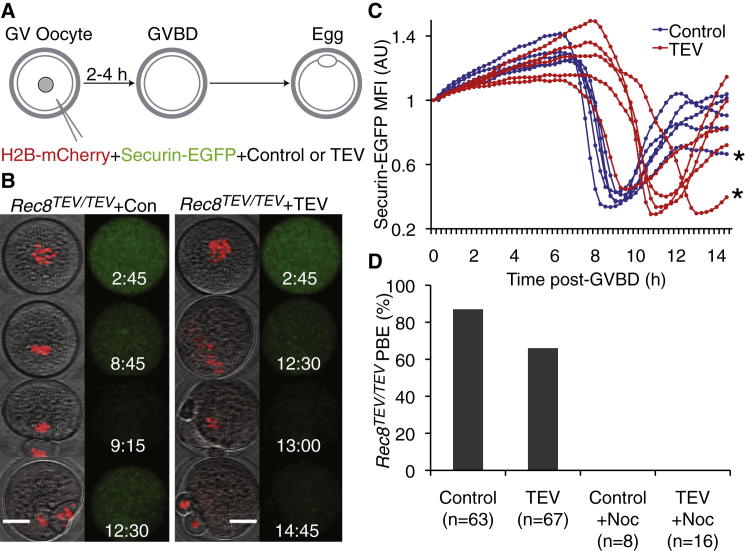
Kinetochores Associated with Individual Chromatids Delay the First Meiotic Division of Oocytes (A) *Rec8*^TEV/TEV^ GV oocytes injected with mRNA encoding H2B-mCherry, securin-EGFP, and frameshift control or wild-type TEV protease were cultured in IBMX and then released to undergo germinal vesicle breakdown (GVBD). (B) Chromosome dynamics were followed by time-lapse confocal microscopy. Still images from representative movies of control and TEV protease-expressing oocytes are shown with GVBD as t = 0 (hr:min). Left panels show differential interference contrast (DIC) and the H2B-mCherry channel pseudocolored in red, and right panels show securin-EGFP pseudocolored in green. Scale bar represents 25 μm. (C) Securin-EGFP mean fluorescence intensity (MFI) of *Rec8*^TEV/TEV^ oocytes expressing frameshift or wild-type TEV protease. Asterisks mark oocytes whose corresponding still images are seen in (B). (D) Polar body extrusion (PBE) of *Rec8*^TEV/TEV^ oocytes expressing frameshift or wild-type TEV protease. Some oocytes were transferred at 5 hr post GVBD into medium supplemented with low concentrations of nocodazole and cultured in this medium for 15 hr.

We previously reported the destruction of all sister chromatid cohesion in meiosis I oocytes by microinjection of TEV protease into oocytes from *Rec8*^TEV/TEV^ mice that express Rec8 containing TEV protease recognition sites [21], which converts bivalent chromosomes into chromatids. To image the effect on cell-cycle progression, we harvested fully grown germinal vesicle (GV)-stage *Rec8*^TEV/TEV^ oocytes in the presence of phosphodiesterase inhibitor 3-isobutyl-1-methylxanthine (IBMX) and microinjected them with mRNA encoding TEV protease or frameshift TEV protease, histone H2B-mCherry to mark chromosomes, and securin-EGFP to measure anaphase-promoting complex/cyclosome (APC/C) activity [15] (Figure 1A). In oocytes expressing frameshift TEV, IBMX removal leads to GV breakdown (GVBD), biorientation of bivalents on spindles, a drop in securin-EGFP fluorescence due to APC/C activation, conversion of bivalents into dyad chromosomes, segregation of dyads to opposite poles, and PBE. PBE occurred in 84% of control cells (n = 63) (Figures 1B and 1D). TEV expression in oocytes converted their 20 bivalents to 80 chromatids that moved from one end of the spindle to the other (Figure 1B; see also Figure S1A and Movie S1 available online).

Given that the 40 univalent chromosomes of *Mlh1*^−/−^ oocytes from several mouse strains also move from one end of the spindle to the other and that their failure to biorient is accompanied by an indefinite cell-cycle arrest, we expected that the 80 individual kinetochores of *Rec8*^TEV/TEV^ oocytes injected with TEV protease would generate a similarly robust SAC response. Surprisingly, 62% of oocytes (n = 67) containing only chromatids degraded securin-EGFP after a short delay and divided, producing highly aneuploid eggs (Figures 1B–1D). They frequently produced two polar bodies simultaneously (Figure 1B; Figure S1A; Movie S1), a phenomenon presumably caused by interaction of both poles of the extended spindles with the cell cortex [22]. Similar results were obtained when recombinant TEV protease was injected (data not shown). Crucially, the precocious loss of cohesion merely retarded PBE by 2–3 hr (control PBE 9 hr 22 min ± 47 min [n = 37], TEV protease PBE 11 hr 48 min ± 1 hr 34 min [n = 27], t < 0.0001). This delay depends on the SAC, because a dominant-negative version of the APC/C activator Cdc20 (Cdc20R132A) that cannot be bound by Mad2 [23, 24] advanced PBE of *Rec8*^TEV/TEV^ oocytes (6 hr 19 min ± 1 hr 11 min [n = 26]) when coexpressed with TEV protease. PBE was also advanced by inhibition of Aurora kinase (Figure S1B), suggesting that the delay depends on activity of the chromosomal passenger complex (CPC) controlling the SAC, as in wild-type oocytes [25]. To test whether cohesin is essential for a robust SAC response, we cultured *Rec8*^TEV/TEV^ oocytes expressing TEV or frameshift TEV protease in low concentrations of nocodazole after spindle assembly. Neither set of oocytes underwent PBE (Figure 1D), suggesting that microtubule depolymerization can trigger a robust SAC response in the absence of cohesin. These observations suggest that kinetochores associated with chromatids activate the SAC in a CPC-dependent manner during meiosis I but do so in a less robust manner than those associated with univalent chromosomes. During the course of this work, it was reported that strain background affects the cell-cycle arrest caused by Mlh1 loss [19]. As will become apparent from experiments described below, the more robust SAC response associated with univalents as compared to chromatids cannot be attributed to strain background differences.

### SAC Response to Kinetochores Associated with Chromatids in Mitotic Zygotes

**Figure 2:**
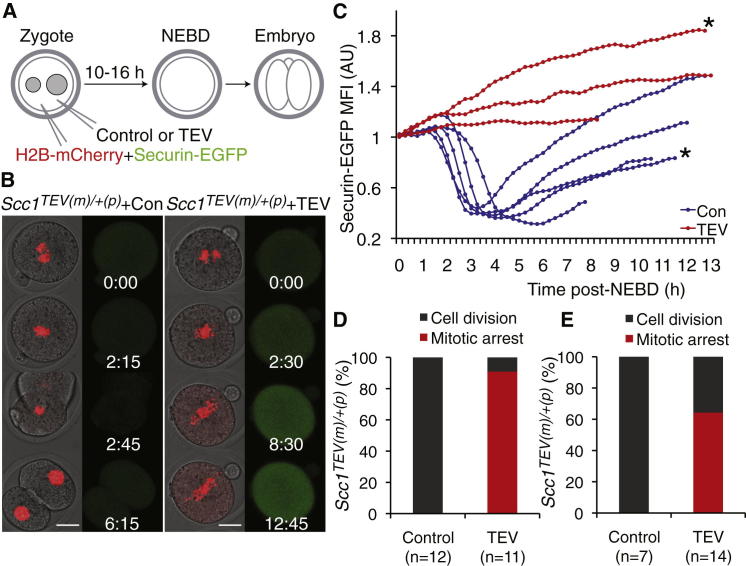
Kinetochores Associated with Individual Chromatids Trigger a Mitotic Arrest in Zygotes (A) Pronuclear-stage *Scc1*^TEVMyc(m)/+(p)^ (*Scc1*^TEV(m)/+(p)^) zygotes were injected during interphase with buffer or TEV protease and mRNA encoding H2B-mCherry and securin-EGFP and imaged for 24 hr. (B) Still images from representative movies of control and TEV protease-injected *Scc1*^TEVMyc(m)/+(p)^ zygotes shown with nuclear envelope breakdown (NEBD) as t = 0 (hr:min). Left panels show DIC and the H2B-mCherry channel pseudocolored in red, and right panels show securin-EGFP pseudocolored in green. Scale bar represents 25 μm. (C) Securin-EGFP fluorescence levels of *Scc1*^TEVMyc(m)/+(p)^ zygotes injected with buffer or TEV protease. Asterisks mark zygotes whose corresponding still images are seen in (B). (D) Cell division of *Scc1*^TEVMyc(m)/+(p)^ zygotes injected with buffer or TEV protease during interphase, as outlined in (A). (E) *Scc1*^TEVMyc(m)/+(p)^ zygotes were injected with mRNA encoding H2B-mCherry and securin-EGFP during interphase. Following NEBD, zygotes in prometaphase were injected with mRNA encoding frameshift or wild-type TEV protease, and cell division was scored up to 17 hr later.

Kinetochores associated with individual chromatids are not a normal feature of meiosis I, but they are a normal feature of mitosis. Mitotic cells might therefore be expected to mount a more vigorous SAC response than meiosis I cells upon loss of sister chromatid cohesion. To test this under comparable conditions, in particular in cells with a similar cellular volume, we analyzed the consequences of using TEV protease to create individual chromatids in fertilized eggs (i.e., zygotes). Since maternally provided Scc1 mediates sister chromatid cohesion during the first embryonic cell cycle, zygotes with TEV-cleavable cohesin can be produced by mating *Scc1*^TEVMyc/TEVMyc^ females to *Scc1* males [21]. Pronuclear stage *Scc1*^TEVMyc(m)/+(p)^ zygotes (G1/S phase, 6–10 hr postfertilization) were injected with recombinant TEV protease or buffer and H2B-mCherry and securin-EGFP mRNA (Figure 2A). The timing of entry into mitosis was variable, presumably reflecting differences in fertilization timing in utero. In contrast, the duration of mitosis from nuclear envelope breakdown (NEBD) to anaphase was constant in control zygotes (3 hr 4 min ± 25 min, n = 12). In 12 of 12 control zygotes, chromosome alignment on metaphase plates preceded the drop in securin-EGFP fluorescence, which was followed by sister chromatid disjunction and cell division, producing two-cell embryos (Figure 2B). In 10 of 11 zygotes whose Scc1 had been cleaved by TEV, most if not all chromosomes were converted to chromatids that continually moved from one end of the spindle to the other (Movie S2). Crucially, securin-EGFP fluorescence never dropped, and cells remained in a mitotic state for up to 17 hr (Figures 2B–2D). Thus, precocious loss of sister chromatid cohesion induced by cohesin inactivation during interphase of zygotes triggers a mitotic arrest.

The prolonged arrest of zygotes contrasts with the transient one observed in meiosis I oocytes. A potential caveat is that these different responses might be caused by differences in the timing with which cohesin was cleaved, which occurred during DNA replication in zygotes but long afterward in oocytes. One reason why this might matter is that the arrest of zygotes might be caused by interfering with cohesin functions during interphase that are unrelated to cohesion. To exclude this, we delayed Scc1 cleavage until after cells had entered mitosis. Pronuclear stage *Scc1*^TEVMyc(m)/+(p)^ zygotes were injected with H2B-mCherry and securin-EGFP mRNA and injected with TEV protease mRNA after NEBD. Injection of control mRNA into prometaphase zygotes had no effect on mitotic progression, and 7 of 7 cells divided within 2.5 hr (Figure 2E). Of 14 TEV-injected cells, 5 divided possibly due to insufficient TEV expression and Scc1 cleavage prior to APC/C activation (a period < 2 hr). In contrast, 9 of 14 TEV-injected cells arrested indefinitely in a mitotic state with chromatids and high securin-EGFP levels. These data suggest that chromatids produced by Scc1 cleavage in prometaphase zygotes are sufficient to trigger a stable mitotic arrest. Given that there is a negligible difference in cell volume between oocytes and zygotes, the weak SAC response of oocytes to chromatids cannot be attributed to dilution of MCC in a large cell. Our data instead imply that the SAC is under developmental control. Kinetochores associated with chromatids are much less effective in mounting a robust SAC response in meiosis I oocytes than in zygotes. Current experiments do not allow us to say whether the transition to a state that responds more robustly occurs upon completion of meiosis I or upon fertilization.

### SAC Response to Mono-oriented Kinetochores of Univalents

**Figure 3:**
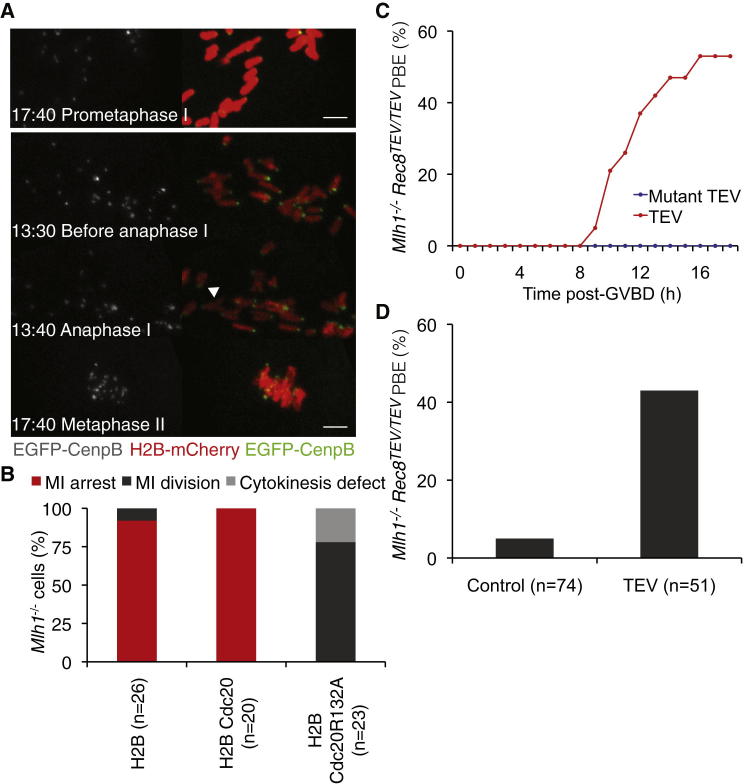
Meiosis I Arrest of *Mlh1*^*−/−*^ Oocytes Depends on the SAC and Cohesin (A) Still images from representative high resolution movies of *Mlh1*^−/−^ oocytes injected with mRNA encoding H2B-mCherry, EGFP-CenpB and Cdc20 (top panel) or Cdc20R132A (lower panels). Time is shown relative to GVBD (t = 0, hr:min). Left panel shows the EGFP-CenpB channel in gray, and right panels show H2B-mCherry and EGFP-CenpB pseudocolored in red and green, respectively. Arrowhead indicates resolved arm cohesion. Scale bar represents 5 μm. (B) PBE of *Mlh1*^−/−^ oocytes expressing H2B-mCherry alone or in combination with either Cdc20 or Cdc20R132A up to 14 hr post GVBD. (C) *Mlh1*^−/−^*Rec8*^TEV/TEV^ GV oocytes injected with mRNA encoding H2B-mCherry and frameshift (n = 10) or wild-type (n = 19) TEV were cultured for 1–2 hr in IBMX, then released to undergo GVBD and scored for PBE. (D) PBE of *Mlh1*^−/−^*Rec8*^TEV/TEV^ oocytes expressing frameshift or wild-type TEV protease up to 14 hr post GVBD.

Given the striking difference in the responses of oocytes with univalents and chromatids, we reinvestigated the behavior of *Mlh1*^−/−^ oocytes. We initially addressed two questions: do we also see an extended arrest, and if so, is this arrest attributable to the SAC? Greater than 90% of *Mlh1*^−/−^ oocytes arrested in meiosis I (n = 26), even when coinjected with wild-type Cdc20 mRNA (n = 20) (Figures 3A and 3B). Importantly, univalent chromosomes contained only a single EGFP-CenpB focus for >17 hr post GVBD (Figure 3A), implying that their kinetochores remain mono-oriented. Like the chromatids induced by Rec8 cleavage, univalents moved back and forth along the spindles (Movie S3), indicating an unstable association between mono-oriented kinetochores and microtubules. Coinjection of Cdc20R132A mRNA had a dramatic effect in 27 of 27 cells, causing univalents to segregate randomly without prior congression to a metaphase plate (Figure 3A), and triggering advanced securin degradation (Figures S2A and S2B). Chromosome segregation occurred in the absence of sister kinetochore biorientation, was accompanied by APC/C activation, and followed by cell division in most cases (Figures 3A and 3B; Figure S2B). The segregation of univalents was accompanied by loss of arm cohesion and was followed by biorientation of sister kinetochores associated with dyad chromosomes on metaphase II spindles (Figure 3A). Interestingly, cytokinesis failed in 25% of oocytes expressing Cdc20R132A (Figure 3B), possibly because the APC/C had been activated prior to migration of the spindle to the cortex. We conclude that the extended, frequently indefinite arrest of *Mlh1*^−/−^ oocytes in meiosis I depends on binding of Mad2 to Cdc20. SAC activity is therefore responsible for inhibiting PBE in *Mlh1*^−/−^ oocytes.

### SAC-Dependent Meiosis I Arrest of Univalents Depends on Cohesin

The simplest explanation for the different behavior of *Mlh1*^−/−^ oocytes and those whose Rec8 has been cleaved by TEV is that cohesin affects the structure of mono-oriented kinetochores on univalent (as well as bivalent) chromosomes in a manner that enhances their ability to generate MCC. If so, then the arrest of *Mlh1*^−/−^ oocytes should be abbreviated by cleaving cohesin. However, there are alternative explanations. What if the SAC response of *Mlh1*^−/−^ oocytes is induced by DNA damage caused by defective resolution of recombination intermediates along chromosome arms? If so, then cleaving cohesin might not alleviate the arrest, unless cohesin itself were required for DNA damage signaling, which is also a possibility [26]. This raises a key issue: is the phenotype caused by Rec8 cleavage epistatic to that caused by *Mlh1*^−/−^, or vice versa?

To answer this, we created *Mlh1*^−/−^ *Rec8*^TEV/TEV^ oocytes in which TEV-cleavable Rec8 maintains univalent cohesion. Because the *Mlh1*^−/−^ *Rec8*^TEV/TEV^ oocytes have a mixed B6/129Sv background, we first confirmed that they do indeed arrest in meiosis I. Importantly, 95% of *Mlh1*^−/−^ *Rec8*^*TEV/TEV*^ oocytes arrested in meiosis I, and only 5% underwent PBE (n = 74) (Figures 3C and 3D). To test whether their arrest depends on cohesin, *Mlh1*^−/−^ *Rec8*^TEV/TEV^ oocytes were coinjected with TEV or frameshift TEV mRNA and scored for PBE (Figure 3C). Strikingly, TEV protease injection triggered chromatid segregation to one pole or another without prior congression to a metaphase plate and PBE in 43% of *Mlh1*^−/−^ *Rec8*^TEV/TEV^ oocytes (n = 51) (Figures 3D; Figure S2C), implying that cohesin is required for their extended meiosis I arrest. Thus, the SAC response associated with Rec8 cleavage is epistatic to that associated with loss of Mlh1.

To address whether cohesin cleavage affects recruitment of SAC components to kinetochores, we analyzed on chromosome spreads the distribution of Bub1. Following conversion of univalents to chromatids due to injection of TEV mRNA into *Mlh1*^−/−^ *Rec8*^TEV/TEV^ oocytes, Bub1 still localized to kinetochores in the absence of cohesin (Figure S3A). Since the SAC is also sustained by Aurora B/C kinase, a CPC subunit, we examined the localization of phosphorylated active Aurora C on chromosome spreads. Aurora C was enriched at kinetochores of bivalents and univalents and still detectable on kinetochores of chromatids (Figure S3B). Together with the finding that chromatids trigger a delay in PBE that depends on Aurora activity (Figure S1B), we conclude that the CPC can function in the absence of cohesin.

### SAC-Dependent Arrest of Univalents Depends on Cohesin near Kinetochores

Since cohesin has been implicated in DNA damage signaling, our finding that the meiosis I arrest of *Mlh1*^−/−^ *Rec8*^TEV/TEV^ oocytes depends on cohesin integrity does not exclude the possibility that their SAC response originates from DNA damage along chromosome arms. According to this scenario, it is cleavage of cohesin along chromosome arms that relieves the arrest. In this case, cleavage of cohesin solely in the vicinity of kinetochores should have little effect. In contrast, selective cleavage at kinetochores should shorten the meiosis I arrest if the SAC signal arises from mono-oriented kinetochores that cannot be brought under tension (Figure S4A). We therefore attempted to localize Rec8 cleavage by targeting active or catalytically inactive (TEVD81N) TEV protease to kinetochores by fusing both proteins to a CenpC motif, which causes association with kinetochores, and mCherry, which enables their visualization. CenpC-mCherry-TEV (CCTEV) colocalized with EGFP-CenpB as single foci at mono-oriented kinetochores in prometaphase I and as split foci associated with bioriented sister kinetochores in metaphase II of wild-type oocytes (Figure S4B).

**Figure 4:**
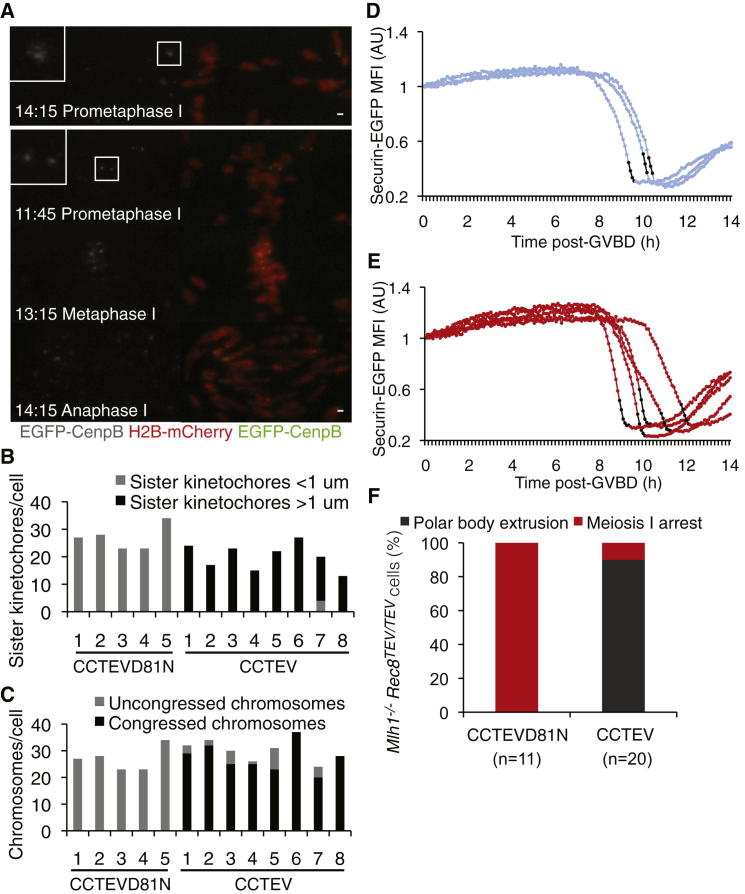
Selective Cleavage of Centromeric Cohesin Relieves the Meiosis I Arrest Triggered by Kinetochores Associated with Univalent Chromosomes (A) *Mlh1*^−/−^*Rec8*^TEV/TEV^ GV oocytes injected with mRNA encoding H2B-mCherry, EGFP-CenpB, and CCTEVD81N (top panel) or CCTEV (lower panels) were cultured for 1–2 hr in IBMX and then released to undergo GVBD. Time is shown relative to GVBD (t = 0, hr:min). Insets display EGFP-CenpB foci in prometaphase I. Scale bar represents 1 μm. (B) Distance between sister kinetochores was determined for CCTEVD81N- and CCTEV-expressing *Mlh1*^−/−^*Rec8*^TEV/TEV^ oocytes. Kinetochore measurements were performed at 17 hr post GVBD for CCTEVD81N-expressing cells, which corresponds to prometaphase since these cells remain arrested in meiosis I. Kinetochore measurements were performed at metaphase I for CCTEV-expressing cells. (C) Chromosome congression was determined by analyzing chromosome location within a 13 × 18 μm box centered on the metaphase I plate. (D) Securin-EGFP fluorescence levels of *Mlh1*^+/+^*Rec8*^TEV/TEV^ oocytes expressing CCTEVD81N and H2B-mCherry, with black time points indicating metaphase until separation of chromosome masses. (E) Securin-EGFP fluorescence levels of *Mlh1*^−/−^*Rec8*^TEV/TEV^ oocytes expressing CCTEV and H2B-mCherry, with black time points indicating metaphase until separation of chromosome masses. (F) PBE of *Mlh1*^−/−^*Rec8*^TEV/TEV^ oocytes expressing CCTEVD81N or CCTEV up to 14 hr post GVBD.

To obtain selective cleavage during meiosis I, we found it necessary to inject CCTEV mRNA with a 10-fold lower concentration. GV-stage *Mlh1*^−/−^ *Rec8*^TEV/TEV^ oocytes were injected with CCTEV or CCTEVD81N, H2B-mCherry, and EGFP-CenpB mRNA followed by time-lapse microscopy (Figure 4A). CCTEVD81N had no discernible effect. All oocytes contained univalent chromosomes that failed to congress to metaphase plates and arrested indefinitely in meiosis I (Figures 4A and 4F). CCTEV, in contrast, clearly induced sister kinetochore splitting, as measured by distinct EGFP-CenpB foci separated by more than 1 μm, without any discernible effect on arm cohesion. Sister kinetochore splitting was accompanied by congression of most chromosomes to a metaphase plate (Figures 4A–4C; Figure S4C). It also induced anaphase chromosome movements and PBE with kinetics similar to wild-type (Figures 4D and 4E; Movie S4). Because cleavage of cohesin only in the vicinity of kinetochores shortened the meiosis I arrest, we conclude that cohesin is required for efficient MCC production, at least in the absence of chiasmata. Our experiment also demonstrates that Rec8-cohesin is necessary for sister kinetochore mono-orientation in oocytes.

## Conclusions

The SAC response of meiosis I oocytes to a few achiasmate or misaligned chromosomes is weak [2–7, 19], giving rise to the notion that there is a threshold amount of congressed chromosomes to satisfy SAC requirements. We describe here the consequences of 80 chromatids whose kinetochores cannot come under tension created by biorientation on MCC production as measured by APC/C activation. To our surprise, we found that the SAC responds differently to precocious loss of sister chromatid cohesion in meiosis I and mitosis. Kinetochores associated with chromatids are far less effective in mounting a robust SAC in meiosis I oocytes than in zygotes, implying that the SAC is developmentally regulated. Current data do not allow us to distinguish whether the change in SAC response is due to differences in chromosome biology or in cytoplasmic factors. However, we have further discovered that cohesin is especially important for SAC activity in meiosis I oocytes. The effect is chromosome specific, as centromeric cohesin shapes kinetochore structure to promote efficient MCC production in meiosis I. A crucial implication for aging oocytes is that cohesin deterioration compromises the SAC in two ways: by promoting sister kinetochore biorientation and by impairing efficient MCC production at kinetochores that have not come under tension, thereby leading to meiosis I chromosome segregation errors such as those underlying trisomy 21 or Down’s syndrome.

## Experimental Procedures

Mice were housed in animal facilities at the University of Oxford, where all procedures were approved by local ethical review committees and licensed by the Home Office under the Animal (Scientific Procedures) Act 1986. Mice were also housed in the animal facility of IMBA, where all experiments were carried out according to valid project licences approved by Austrian veterinary authorities.

### In Vitro Culture and Microinjection of Cells

Fully grown mouse GV oocytes and zygotes were isolated, cultured, and injected as described previously [21]. In experiments where oocytes were cultured in medium containing 400 nM nocodazole or 5 μM ZM447439, controls were treated with an equivalent amount of ethanol or DMSO solvent, respectively. For TEV cleavage experiments in prometaphase zygotes, cells were first injected with mRNA encoding H2B-mCherry and securin-EGFP in interphase and monitored manually for NEBD. Cells were injected with control or TEV protease mRNA within 30 min of NEBD. For experiments with CCTEV or CCTEV81N, oocytes were microinjected with 5–10 pl of 30 ng/μl mRNA for visualization of mCherry localization and with 3 ng/μl mRNA for cleavage of centromeric cohesin.

### Time-Lapse Live Confocal Microscopy

Cells were cultured in a PeCon (Erbach) environmental microscope incubator allowing maintenance of a 5% CO_2_ atmosphere with humidity at 37°C during time-lapse experiments. A customized Zeiss LSM510 META confocal microscope equipped with Plan C-Apochromat 63×/1.2 NA water immersion objective lens was used for image acquisition. For detection of EGFP and mCherry, 488 nm and 561 nm excitation wavelengths and band-pass 505–550 and long-pass 575 filters were used. Chromosomes labeled with H2B-mCherry were tracked with an EMBL-developed tracking macro [27] adapted to our microscope. Image stacks of 7–11 slices of 2–3.4 μm were captured every 5–15 min for up to 18 hr. Quantitative analysis of the density of fluorescence was performed with ImageJ software (http://rsb.info.nih.gov/ij). To measure securin-EGFP signal, we defined the area occupied by the cell and measured mean fluorescence intensity (MFI) of the signal within this area. Values were corrected for background fluorescence and normalized to the value at NEBD or GVBD as described previously [15]. In order to reduce noise in the ratio differences, the securin data were smoothed by calculating the average of four time points.

## Supplemental Information

Document S1. Figures S1–S4 and Supplemental Experimental Procedures

Movie S1. *Rec8*^TEV/TEV^ Oocyte Expressing H2B-mCherry, Securin-EGFP, and TEV Protease Undergoes the First Meiotic Division with a Delay, Related to Figure 1 Time is relative to germinal vesicle breakdown (t = 0, hr:min).

Movie S2. *Scc1*^TEVMyc(m)/+(p)^ Zygote Expressing H2B-mCherry, Securin-EGFP, and TEV Protease Arrests in Mitosis, Related to Figure 2 Time starts in interphase (hr:min).

Movie S3. *Mlh1*^−/−^ Oocyte Expressing H2B-mCherry and Securin-EGFP Arrests in Meiosis I, Related to Figure 3

Movie S4. *Mlh1*^−/−^ Rec8^TEV/TEV^ Oocyte Expressing CCTEV, H2BmCherry, and EGFP-CenpB Undergoes the First Meiotic Division, Related to Figure 4
