# Supplementary material for: Spindle Assembly Checkpoint of Oocytes Depends on a Kinetochore Structure Determined by Cohesin in Meiosis I
Source: Curr Biol. 2013 Dec 16;23(24):2534–9. doi: 10.1016/j.cub.2013.10.052 (PMC3898714; doi:10.1016/j.cub.2013.10.052)
Supplement: Document S1. Figures S1–S4 and Supplemental Experimental Procedures [file mmc1.pdf]

Current Biology, Volume *23*

Supplemental Information

Spindle Assembly Checkpoint of Oocytes Depends on a Kinetochore Structure Determined by Cohesin in Meiosis I

Kikuë Tachibana-Konwalski, Jonathan Godwin, Máté Borsos, Ahmed Rattani, David J. Adams, and Kim Nasmyth

Inventory of Supplemental Information

Figure S1. Supports Figure 1 by showing that chromatids cause a delay in polar body extrusion (PBE) that depends on both SAC and CPC activity in oocytes. The delay in PBE is visualized in Movie S1.

Figure S2. Provides additional information for Figure 3 by showing still images of *Mlh1-/-* oocytes expressing Cdc20 or Cdc20R132A. Movie S3 provides an example of an *Mlh1-/-* oocyte arresting in meiosis I.

Figure S3. Further supports Figure 3 by providing evidence that SAC and CPC components can be recruited to kinetochores of univalents and chromatids.

Figure S4. Provides additional information for Figure 4. Figure S4A explains the rationale behind the experiment in Figure 4 by providing models of where the cohesin-dependent checkpoint signal might originate. Figure S4B shows the control experiment that CCTEV localizes to kinetochores. Figure S4C demonstrates how chromosome congression in Figure 4C was determined.

Supplemental Movie Legends

Supplemental Experimental Procedures

Supplemental References

Figure S1

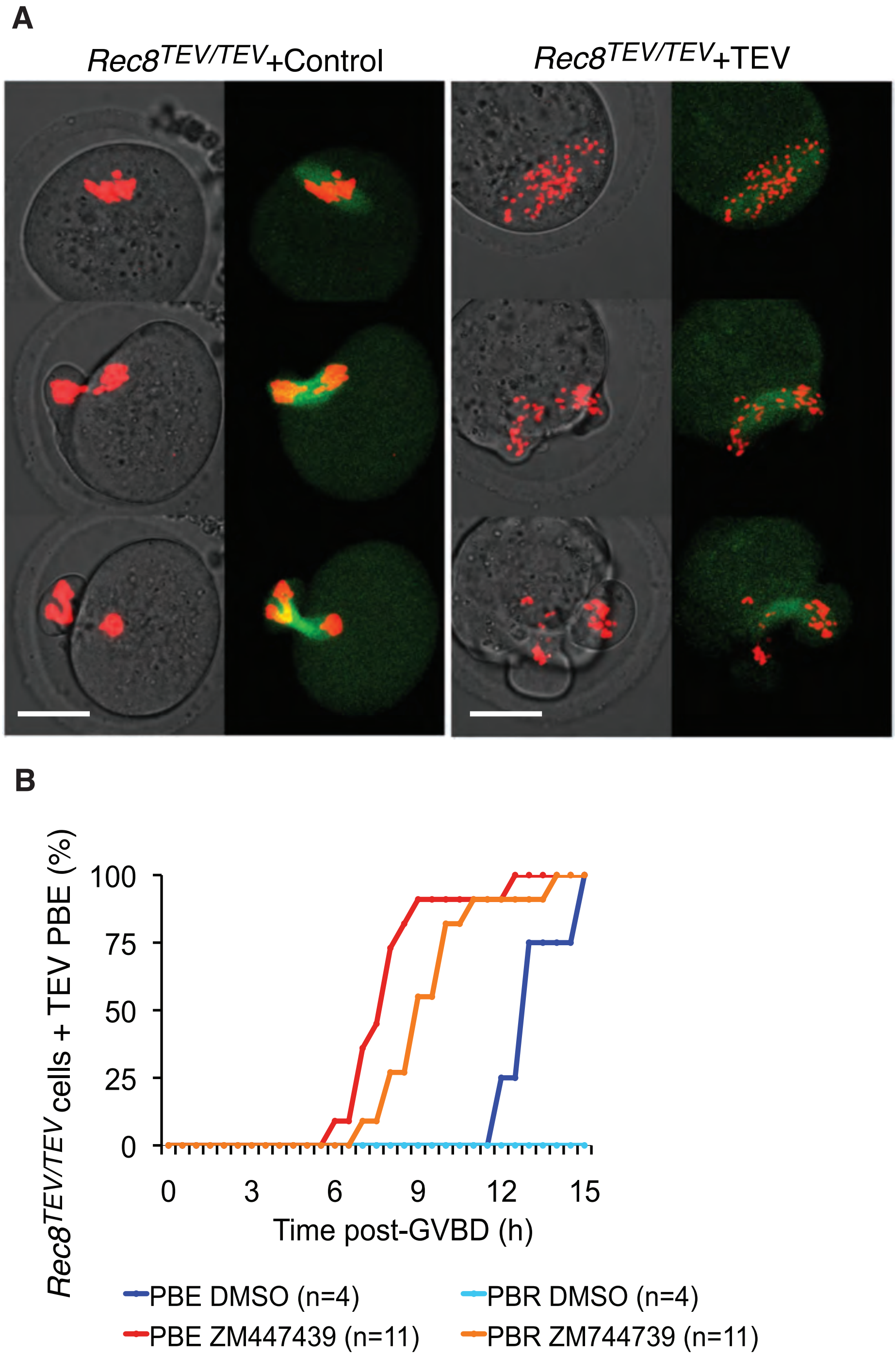
A
Rec8TEV/TEV+Control
Rec8TEV/TEV+TEV
B
Rec8TEV/TEV cells + TEV PBE (%)
100
75
50
25
0
0
3
6
9
12
15
Time post-GVBD (h)
PBE DMSO (n=4)
PBR DMSO (n=4)
PBE ZM447439 (n=11)
PBR ZM744739 (n=11)

Figure S2

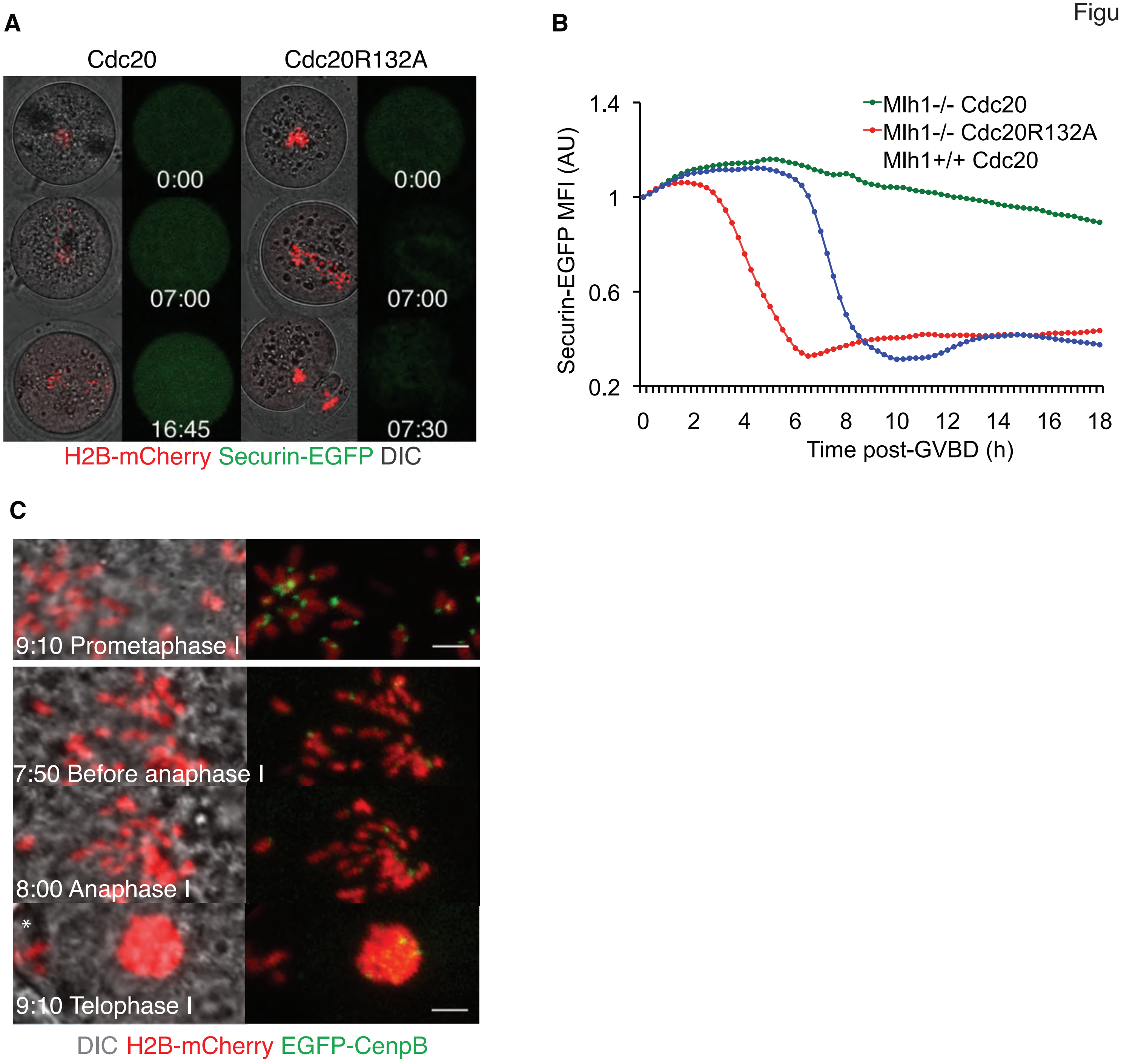
A
Cdc20
Cdc20R132A
0:00
07:00
16:45
0:00
07:00
07:30
H2B-mCherry Securin-EGFP DIC
B
Mlh1-/- Cdc20
Mlh1-/- Cdc20R132A
Mlh1+/+ Cdc20
Securin-EGFP MFI (AU)
1.4
1
0.6
0.2
0
2
4
6
8
10
12
14
16
18
Time post-GVBD (h)
C
9:10 Prometaphase I
7:50 Before anaphase I
8:00 Anaphase I
*
9:10 Telophase I
DIC H2B-mCherry EGFP-CenpB

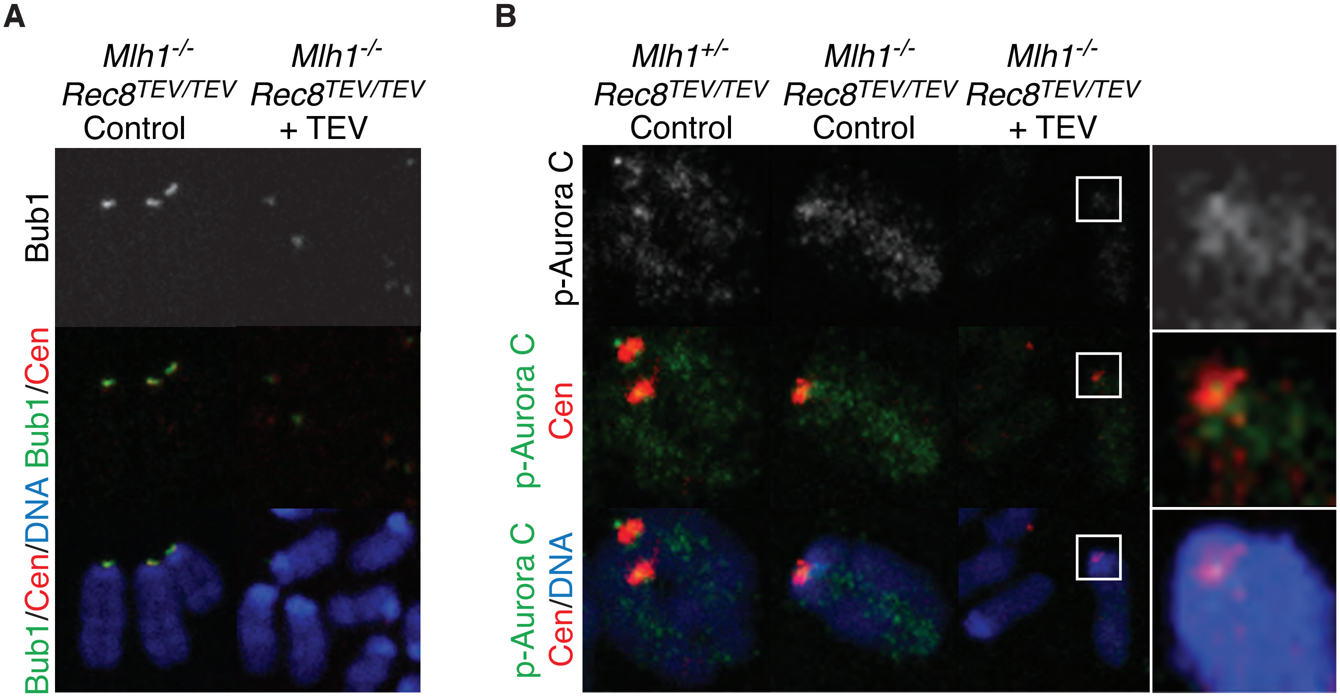
A
Mlh1-/- Rec8TEV/TEV Control
Mlh1-/- Rec8TEV/TEV + TEV
Bub1
Bub1/Cen
Bub1/Cen/DNA
B
Mlh1+/- Rec8TEV/TEV Control
Mlh1-/- Rec8TEV/TEV Control
Mlh1-/- Rec8TEV/TEV + TEV
p-Aurora C
p-Aurora C Cen
p-Aurora C Cen/DNA

Figure S4

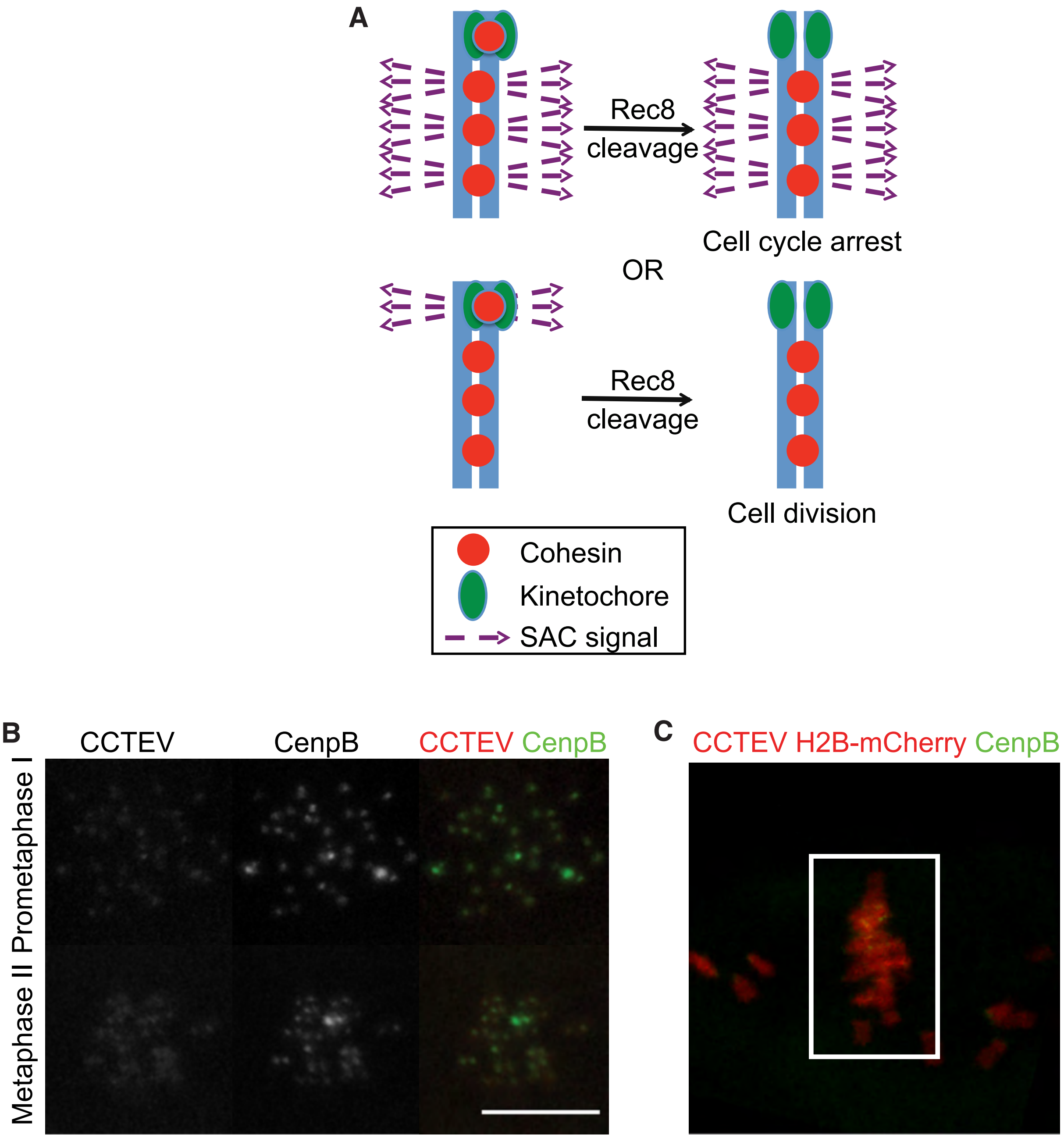
A
Rec8
cleavage
Cell cycle arrest
OR
Rec8
cleavage
Cell division
Cohesin
Kinetochore
SAC signal
B
CCTEV
CenpB
CCTEV CenpB
Prometaphase I
Metaphase II
C
CCTEV H2B-mCherry CenpB

Supplementary Figure Legends

Figure S1, related to Figure 1: Chromatids trigger spindle pole elongation and delay PBE in a CPC-dependent manner

(A) *Rec8*$^{TEV/TEV}$ oocytes expressing H2B-mCherry, Tubulin-EGFP and frameshift or wild-type TEV protease were cultured for 1-2 h in IBMX and released to undergo GVBD (t=0, h:mm). Chromosome movements were visualized by time-lapse confocal microscopy. Still images from representative movies of control and TEV protease expressing oocytes are shown. Left panels show DIC and the H2B-mCherry channel pseudocolored in red and right panels show the Tubulin-EGFP channel and the H2B-mCherry channel pseudocolored in green and red, respectively. Bar, 25 μm.

(B) *Rec8*$^{TEV/TEV}$ oocytes expressing TEV protease were cultured after GVBD in DMSO or the Aurora kinase inhibitor ZM447439. Since inhibition of Aurora kinase also affects cytokinesis, oocytes were scored both for PBE and polar body retraction (PBR) due to failure of cytokinesis.

Figure S2, related to Figure 3: The meiosis I arrest of *Mlh1*$^{-/-}$ oocytes depends on the SAC and cohesin.

(A) *Mlh1*$^{-/-}$ oocytes expressing H2B-mCherry, Securin-EGFP and Cdc20 or Cdc20R132A. The left panels show DIC and the H2B-mCherry channel pseudocolored in red and the right panels show the Securin-EGFP channel pseudocolored in green.

(B) *Mlh1*$^{-/-}$ *and Mlh1*$^{+/+}$ GV oocytes injected with mRNA encoding H2B-mCherry, Securin-EGFP and Cdc20 or Cdc20R132A were cultured for 2 h and then released to undergo GVBD. Securin-EGFP MFI curves representing the means of 6 *Mlh1*$^{-/-}$ cells expressing Cdc20, 4 *Mlh1*$^{-/-}$ cells expressing Cdc20R132A and 6 *Mlh1*$^{+/+}$ cells expressing Cdc20.

(C) *Mlh1*$^{-/-}$ *Rec8*$^{TEV/TEV}$ GV oocytes injected with mRNA encoding H2B-mCherry, EGFP-CenpB and frameshift (top panel) or wild-type TEV (lower panels) were cultured for 1-2 h in IBMX and then released to undergo GVBD. Left panel shows DIC and H2B-mCherry pseudocolored in red and right panels show H2B-mCherry and EGFP-CenpB pseudocolored in red and green, respectively. White star marks the polar body. Bar, 5 μm.

Figure S3, related to Figure 3: Bub1 and Aurora C localize to kinetochores in the absence of cohesin.

(A) Chromosome spreads were prepared of *Mlh1*$^{-/-}$ *Rec8*$^{TEV/TEV}$ meiosis I oocytes expressing frameshift or wild-type TEV protease at 4.5 h post-GVBD. Chromosome spreads were stained with Bub1 antibody (green), CREST to mark centromeres (red) and Hoechst to visualize DNA (blue).

(B) Chromosome spreads were prepared of *Mlh1*$^{+/-}$ *Rec8*$^{TEV/TEV}$ and *Mlh1*$^{-/-}$ *Rec8*$^{TEV/TEV}$ meiosis I oocytes expressing control or wild-type TEV protease at 4.5 h post-GVBD. Chromosome spreads were stained with phospho-Aurora C antibody (green), CREST to mark centromeres (red) and Hoechst to visualize DNA (blue). Inserts displayed on the right show signals increased in brightness for all three channels.

Figure S4, related to Figure 4: Centromeric cohesin is required for efficient MCC production in meiosis I.

(A) Model (upper): If the signal that stops cell cycle progression originates from defective resolution of recombination intermediates on chromosome arms, then cleaving centromeric cohesin should not relieve the meiosis I arrest triggered by univalents. Model (lower): If the signal that stops cell cycle progression originates from mono-oriented kinetochores that cannot be brought under tension created by bi-

orientation, then cleaving cohesin near centromeres would be expected to relieve the meiosis I arrest triggered by univalents.

(B) Wild-type mouse oocyte expressing CCTEV and EGFP-CenpB channels pseudocolored in red and green, respectively. Representative still images for prometaphase I (upper panel) and metaphase II (lower panel) are shown. Bar, 5 µm.

(C) Chromosome congression in Figure 5C was determined by analyzing chromosome location within a 13 µm x 18 µm box centered on the metaphase plate.

Movie S1, related to Figure 1: *Rec8*$^{TEV/TEV}$ oocyte expressing H2B-mCherry, Securin-EGFP and TEV protease undergoes the first meiotic division with a delay. Time is relative to GVBD (t=0, h:mm).

Movie S2, related to Figure 2: *Scc1*$^{TEVMyc(m)/+(p)}$ zygote expressing H2B-mCherry, Securin-EGFP and TEV protease arrests in mitosis. Time starts in interphase (h:mm).

Movie S3, related to Figure 3: *Mlh1*$^{-/-}$ oocyte expressing H2B-mCherry and Securin-EGFP arrests in meiosis I.

Movie S4, related to Figure 5: *Mlh1*$^{-/-}$ *Rec8*$^{TEV/TEV}$ oocyte expressing CCTEV, H2B-mCherry and EGFP-CenpB undergoes the first meiotic division.

Supplemental Experimental Procedures

Mouse strains

Mice were housed in animal facilities at the University of Oxford and all procedures were approved by local Ethical Review Committees and licensed by the Home Office under the Animal (Scientific Procedures) Act 1986. Mice were also housed in the

animal facility of IMBA and all experiments were carried out according to valid project licences, which were approved by the Austrian Veterinary Authorities. Generation of *Rec8*^{e10TEV} (*Rec8*^{TEV}) and *Scc1*^{e11TEVMyc} (*Scc1*^{TEVMyc}) mice has been described previously [1]. *Rec8*$^{TEV/TEV}$ mice on a C57BL6/129Sv background were bred to *Mlh1*$^{+/-}$ mice on a BL6 background [2] to generate *Rec8*$^{TEV/+}$ *Mlh1*$^{+/-}$ mice, which were backcrossed to *Rec8*$^{TEV/TEV}$ to generate *Mlh1*$^{+/-}$ *Rec8*$^{TEV/TEV}$ mice. *Mlh1*$^{+/-}$ *Rec8*$^{TEV/TEV}$ intercrosses produced *Mlh1*$^{-/-}$ *Rec8*$^{TEV/TEV}$ females used for experiments.

Constructs

In vitro transcription vector pRNA [3] was modified by inserting cDNA for Cdc20, Cdc20R132A or frameshift TEV protease into the *Nhe*I/*Not*I cloning site. Similarly, a triple fusion consisting of the C-terminus of mouse CenpC, mCherry and either TEV protease or catalytically dead TEV protease N81D was inserted into the *Nhe*I/*Not*I cloning site. Capped mRNAs were synthesised by *in vitro* transcription using a mMessage mMachine T3 kit (Ambion, Austin, Texas, USA) and purified with RNeasy columns (QIAGEN, Crawley, UK).

Immunofluorescent staining of chromosome spreads

Chromosome spreads were performed as previously described [1]. CREST serum (gift of A. Kromminga, Hamburg, Germany) for marking centromeres, Bub1 (Abcam) and phospho-Aurora C (Bethyl Labs, UK) were used as primary antibodies, and appropriate secondary antibodies conjugated with Alexa Fluor 488 or 597 (Molecular Probes) were used. Images were captured with a Zeiss LSM510 or LSM780 confocal microscope equipped with a 63x oil objective.

Supplemental References

1. Tachibana-Konwalski, K., Godwin, J., van der Weyden, L., Champion, L., Kudo,

N.R., Adams, D.J., and Nasmyth, K. (2010). Rec8-containing cohesin maintains bivalents without turnover during the growing phase of mouse oocytes. Genes Dev. *24*, 2505-2516.

2. Baker, S.M, Plug, A.W., Prolla, T.A., Bronner, C.E., Harris, A.C., Yao, X., Christie, D.M., Monell, C., Arnheim, N., Bradley, A. et al. (1996). Involvement of mouse Mlh1 in DNA mismatch repair and meiotic crossing over. Nat. Genet. *13*, 336-342.

3. McGuinness, B.E., Anger, M., Kouznetsova, A., Gil-Bernabé, A.M., Helmhart, W., Kudo, N.R., Wuensche, A., Taylor, S., Hoog, C., Novak, B,. et al. (2009). Regulation of APC/C activity in oocytes by a Bub1-dependent spindle assembly checkpoint. Curr. Biol. *19*, 369-380.
