# Supplementary figures and images for: Spindle Assembly Checkpoint of Oocytes Depends on a Kinetochore Structure Determined by Cohesin in Meiosis I
Source: Curr Biol. 2013 Dec 16;23(24):2534–9. doi: 10.1016/j.cub.2013.10.052 (PMC3898714; doi:10.1016/j.cub.2013.10.052)

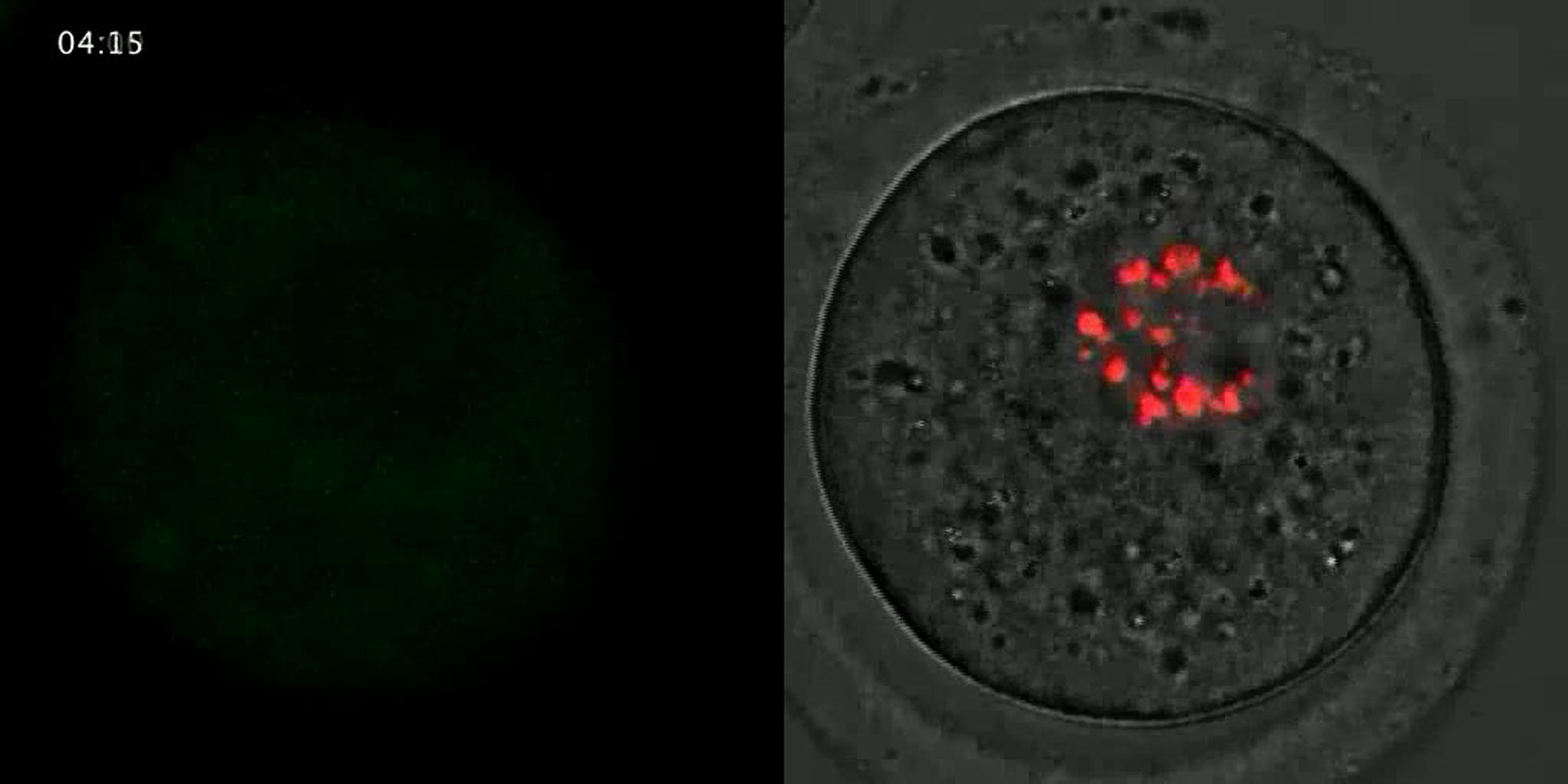

Supplement: Movie S1. Rec8TEV/TEV Oocyte Expressing H2B-mCherry, Securin-EGFP, and TEV Protease Undergoes the First Meiotic Division with a Delay, Related to Figure 1 — Time is relative to germinal vesicle breakdown (t = 0, hr:min). [file mmc2.jpg]

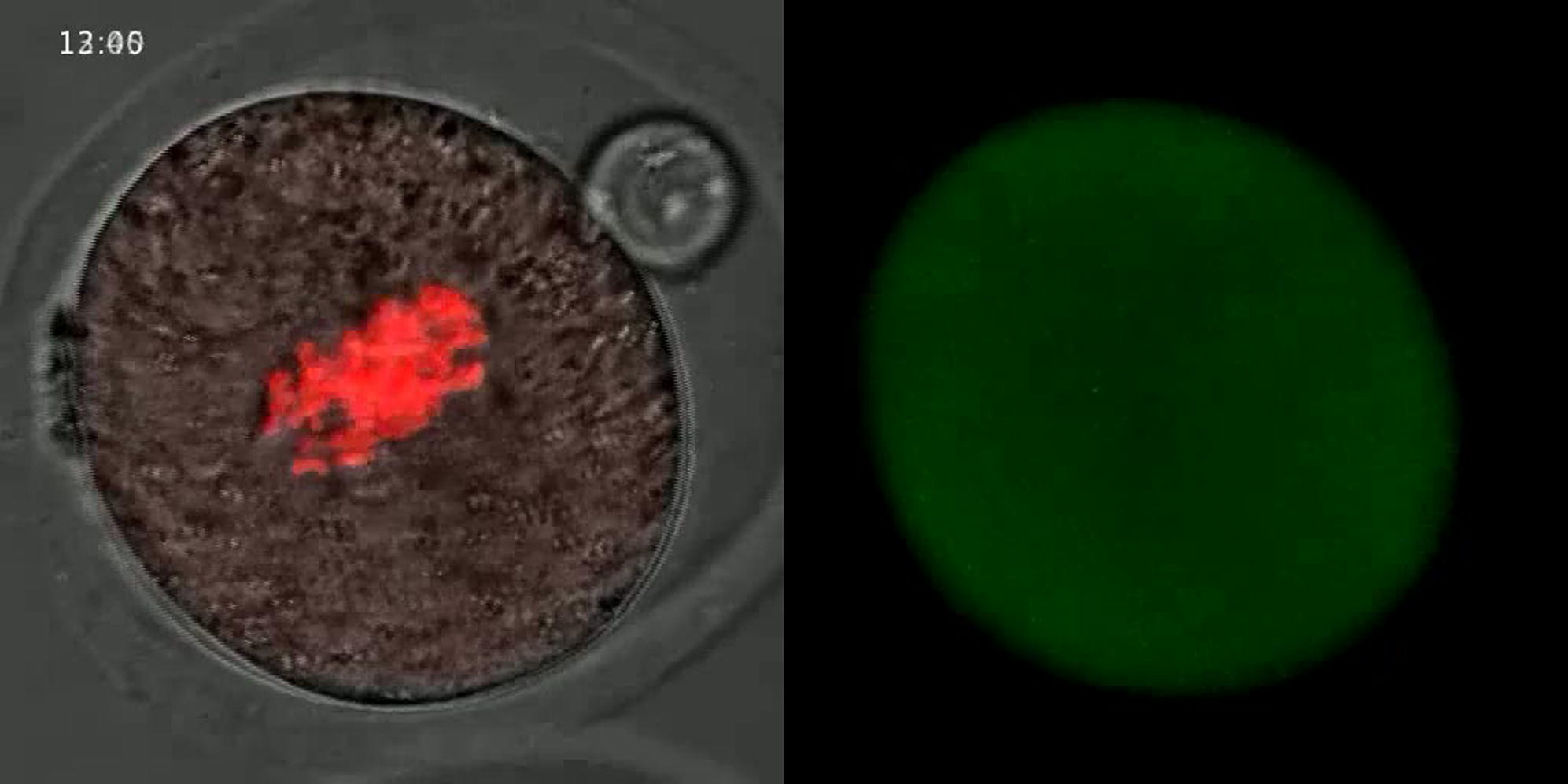

Supplement: Movie S2. Scc1TEVMyc(m)/+(p) Zygote Expressing H2B-mCherry, Securin-EGFP, and TEV Protease Arrests in Mitosis, Related to Figure 2 — Time starts in interphase (hr:min). [file mmc3.jpg]

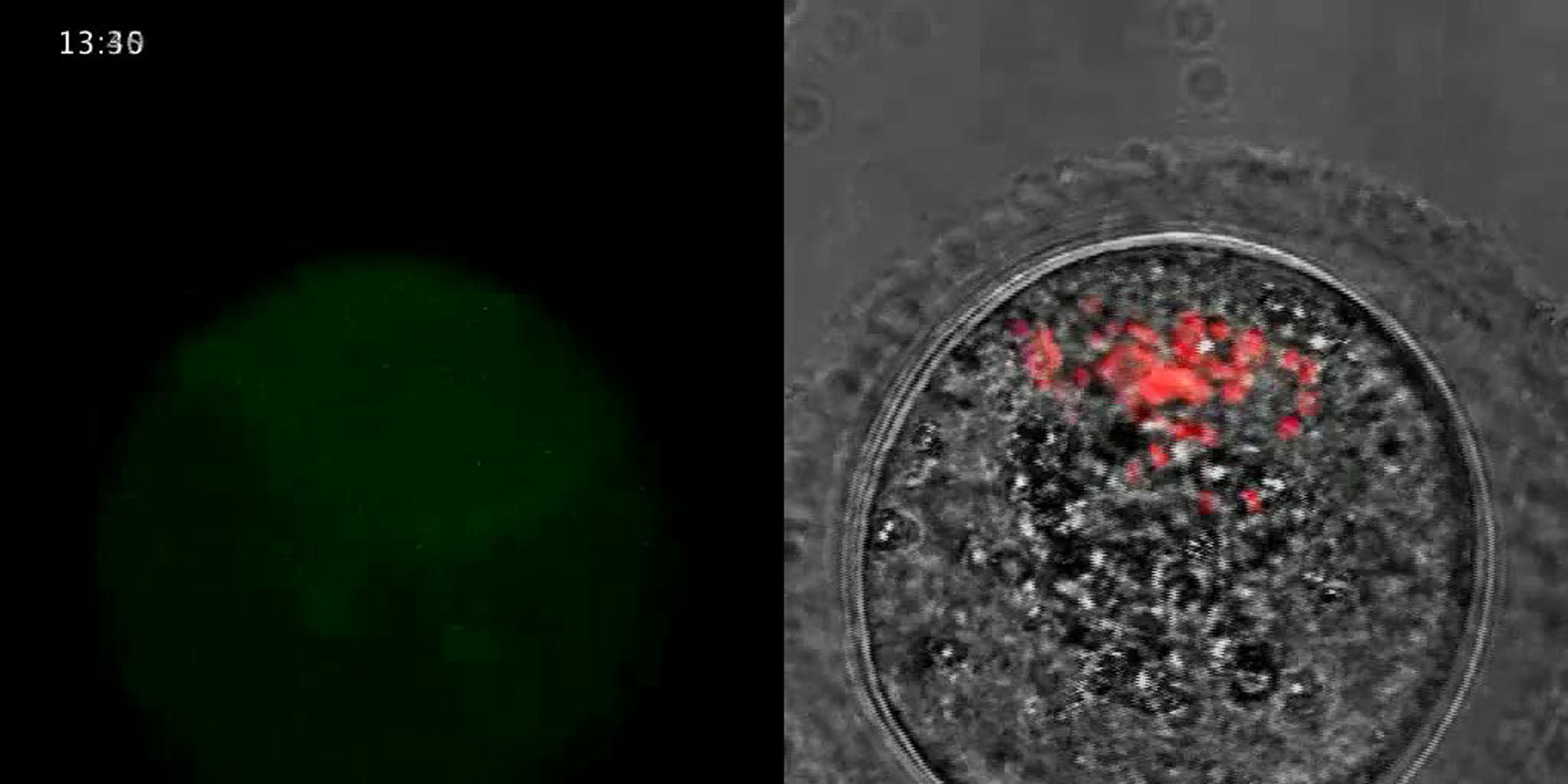

Supplement: Movie S3. Mlh1−/− Oocyte Expressing H2B-mCherry and Securin-EGFP Arrests in Meiosis I, Related to Figure 3 [file mmc4.jpg]

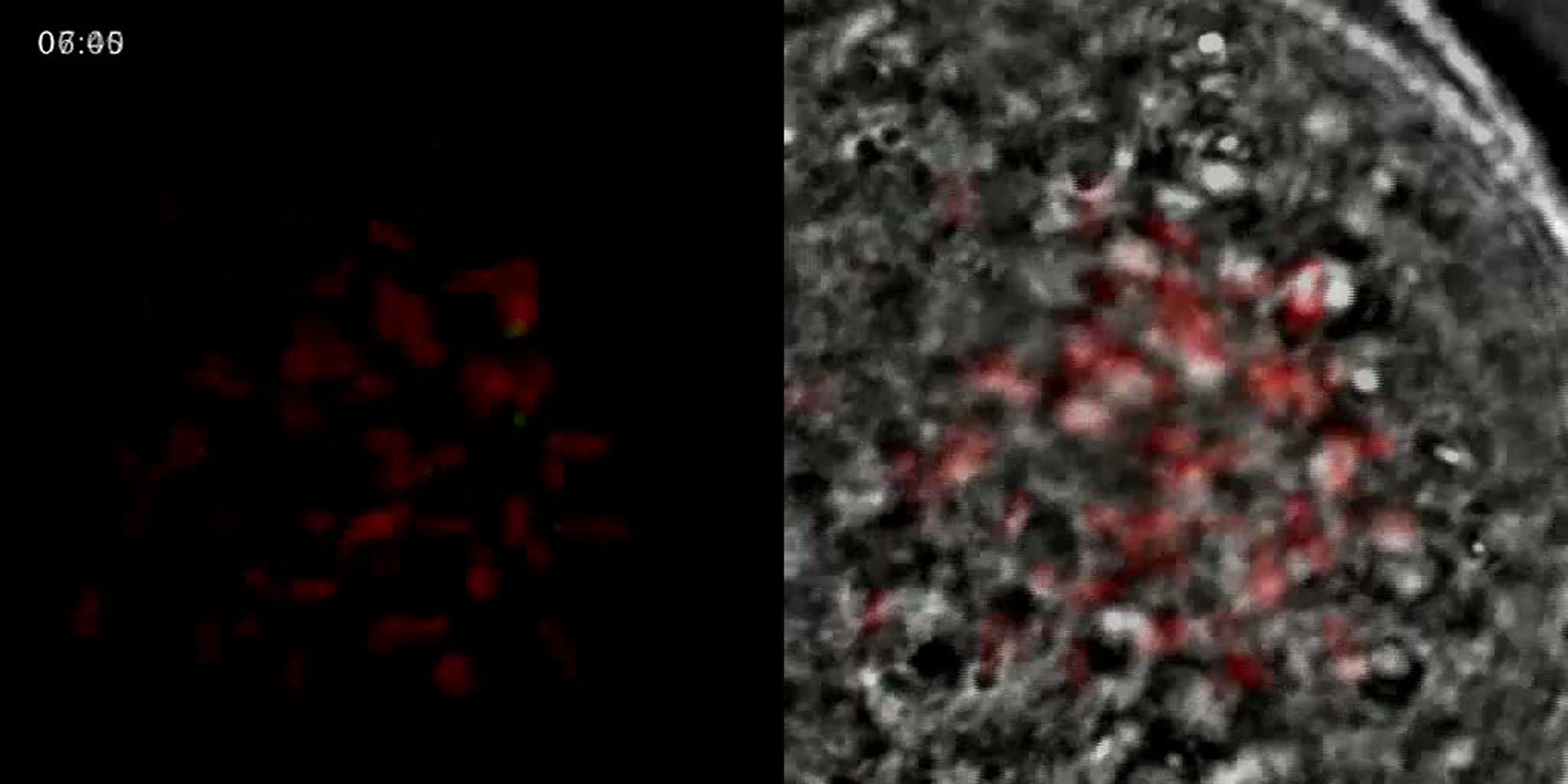

Supplement: Movie S4. Mlh1−/− Rec8TEV/TEV Oocyte Expressing CCTEV, H2BmCherry, and EGFP-CenpB Undergoes the First Meiotic Division, Related to Figure 4 [file mmc5.jpg]
